# Conditional spatial biased intuitionistic clustering technique for brain MRI image segmentation

**DOI:** 10.3389/fncom.2024.1425008

**Published:** 2024-06-28

**Authors:** Jyoti Arora, Ghadir Altuwaijri, Ali Nauman, Meena Tushir, Tripti Sharma, Deepali Gupta, Sung Won Kim

**Affiliations:** ^1^MSIT, New Delhi, India; ^2^Department of Computer Engineering, College of Computer and Information Sciences, Majmaah University, Majmaah, Saudi Arabia; ^3^School of Computer Science and Engineering, Yeungnam University, Gyeongsan, Republic of Korea; ^4^Chitkara University Institute of Engineering and Technology, Chitkara University, Punjab, India

**Keywords:** fuzzy C-means, intuitionistic method, conditional spatial fuzzy C-means, MRI images, segmentation

## Abstract

In clinical research, it is crucial to segment the magnetic resonance (MR) brain image for studying the internal tissues of the brain. To address this challenge in a sustainable manner, a novel approach has been proposed leveraging the power of unsupervised clustering while integrating conditional spatial properties of the image into intuitionistic clustering technique for segmenting MRI images of brain scans. In the proposed technique, an Intuitionistic-based clustering approach incorporates a nuanced understanding of uncertainty inherent in the image data. The measure of uncertainty is achieved through calculation of hesitation degree. The approach introduces a conditional spatial function alongside the intuitionistic membership matrix, enabling the consideration of spatial relationships within the image. Furthermore, by calculating weighted intuitionistic membership matrix, the algorithm gains the ability to adapt its smoothing behavior based on the local context. The main advantages are enhanced robustness with homogenous segments, lower sensitivity to noise, intensity inhomogeneity and accommodation of degree of hesitation or uncertainty that may exist in the real-world datasets. A comparative analysis of synthetic and real datasets of MR brain images proves the efficiency of the suggested approach over different algorithms. The paper investigates how the suggested research methodology performs in medical industry under different circumstances including both qualitative and quantitative parameters such as segmentation accuracy, similarity index, true positive ratio, false positive ratio. The experimental outcomes demonstrate that the suggested algorithm outperforms in retaining image details and achieving segmentation accuracy.

## 1 Introduction

One of the core issues in clinical research methods is to segment the MRI image of human brain. The segmented image helps to detect different diseases related to the brain. Due to the intricate structure of MRI brain images and use of the inherent imaging mechanism includes the presence of noise, delineation of the image boundaries and many other challenges in the segmentation of these MRI images. In literature, several image segmentation methods can be categorized as thresholding (Suzuki and Toriwaki, 1991), region growing, level set methods (Li et al., 2011), model-based methods (Blahova et al., 2023) and unsupervised clustering. Clustering, a primary technique in unsupervised learning, involves grouping a set of patterns into clusters, which can take the form of hard or soft clustering. Soft clustering is preferred over hard clustering due to its ability to assign each pixel varying degrees of membership across all clusters (Li et al., 2022). Fuzzy C-means (FCM), initially given by Dunn (1973) and refined by Bezdek (1981), is a prominent algorithm. The FCM method proves less effective in handling noisy images, primarily because of its vulnerability to noise. The absence of spatial membership matrix in FCM leads to unreliable out-comes in its results.

To address the noise issue, several improved versions of FCM have been proposed (Chen and Zhang, 2004; Wang et al., 2004; Krindis and Chatzis, 2010), which use the image’s local spatial and grayscale information and prove to give better results in segmentation of MRI images. The FCM was altered by Ahmed et al. (2002) adding neighborhood information in the membership matrix. The refinement of similarity metrics incorporates information from all pixels closer to the cluster center, provided they lie within the spatial window and homogeneous region. However, this algorithm exhibits sensitivity to randomly defined initial cluster centers and an associated increase in computational complexity. Pedrycz (1996) proposed modified FCM algorithm by integrating an auxiliary variable and data attributes into the clustering process. By taking into account the domain in a feature space and the values deduced from a particular conditional variable, this method uncovers unique patterns within a set of patterns.

A conditional or auxiliary factor-guided conditional spatial fuzzy C-means (csFCM) methodology was given by Adhikari et al. (2015). This method introduces local spatial interactions among neighboring pixels through a fuzzy weighted membership function. Its advantages include defining more homogenous segments compared to other methods, robust to noise and the elimination of spurious blobs. But the Fuzzy clustering technique does not incorporate measure of the uncertainty degree that are inherent in the image datasets.

A rapid generalized FCM for image segmentation given by Cai et al. (2007), wherein the similarity metric integrates spatial and gray-level details to generate an image with a sum of weights that operates non-linearly. In a similar way, Yang and Zhang (2011) presented a novel penalized FCM, where the penalty term functions as a reconfigure within the algorithm, drawing inspiration from neighborhood maximization. In the literature, number of Fuzzy based segmentation algorithms are proposed for understanding of the anatomical and the functional aspects of the MRI brain images. The segmentation of these MRI images provides a crucial theoretical basis for the analysis and treatment of various brain ailments (Ren et al., 2019). Conventional clustering algorithms such as FCM failed to give the accurate results (Ahmed et al., 2002). To over-come these issues, Yang et al. introduced kernel-based clustering approach embedded with spatial information to violate the effect of noise for the task of segmentation of images (Yang and Tsai, 2008). Chaira (2011) introduced the Intuitionistic based fuzzy approach, which integrates entropy function along with intuitionistic theory for the segmentation of medical images. Notably, IFCM (Intuitionistic Fuzzy C-means) exhibits reduced sensitivity to outliers compared to fuzzy clustering methods. Integrated approaches of clustering were proposed to improve the accuracy of MRI image segmentation. Caldairou et al. (2011) have proposed integrated approach of FCM with the non-local information related to image with the aim of image restoration. Dubey and Mushrif (2016) have investigated different approaches of unsupervised clustering techniques for this purpose. Singh et al. (2024) proposed kernel based FCM clustering approach with bias correction for segmenting of MRI brain images. In this approach image is pre-processed using LZM based filtering and further segmented using kernelized approach of clustering.

The problem with different clustering approaches is the overhead of defining different parameters which is not an easy task. Since medical images are not linearly separable, these clustering techniques are not able to achieve high segmentation accuracy. The majority of these techniques performs severely due to the imperfection of the devices through which image is acquired, poor magnetic field, and other image artifacts.

Besides being susceptible to noise, another significant challenge in MRI image segmentation involves addressing the ambiguity inherent in pixel values. To handle this type of problem, intuitionistic-based clustering algorithms are used, which consist of intuitionistic membership degree characterized by a hesitation degree. Furthermore, by integrating conditional spatial functions into the segmentation framework, the algorithm gains the ability to adapt its smoothing behavior based on the local context of the image with the intuitionistic membership matrix giving more weight to the pixels with more similarity. The intuitionistic based clustering algorithms have been proved to lead novel perspectives in computer vision and therefore, various domains of image segmentation (Vlachos and Sergiadis, 2005; Arora and Tushir, 2019; Thao et al., 2019; Chen et al., 2021) have witnessed the application of clustering algorithms grounded in Intuitionistic metrics.

In this research paper, we introduce an intuitionistic based clustering method integrated with the spatial properties of the image which is guided by the weighted conditional factor termed as Conditional Spatial Intuitionistic Fuzzy C-means (csIFCM) for segmenting MRI brain images. Furthermore, the proposed approach makes noticeable advancements by presenting more adaptable solution toward the segmentation of MRI images. Accurate segmentation of medical image plays a pivotal role in different fields of medical applications, ranging from diagnosis of disease to planning of treatment. Accurate MRI brain image segmentation facilitates in-depth examination of anatomical features and pathological anomalies in clinical research. Therefore, highlighting the wide practical utility of the proposed approach is crucial for facilitating its adoption and integration into existing medical workflows. The primary contributions of this research are outlined below:

- The images are pre-processed using the process of normalization in order to bring the values of the pixels into more conventional form (Internet Brain Segmentation Repository, 2024).
- The proposed approach integrates spatial properties of the image guided by the weighted conditional factor into the membership matrix of the intuitionistic approach of the clustering.
- This algorithm demonstrates its effectiveness by producing robust results with better segmentation accuracy even when faced with challenges such as noise and intensity inhomogeneity.
- The proposed model’s performance was evaluated using a range of standard metrics and the results obtained confirms the outstanding performance over the existing techniques.

The subsequent sections are structured as follows: Section 2 delves into the existing literature concerning the FCM algorithm, csFCM, and IFCM algorithms. Section 3 provides the detailed methodology about the proposed work. The experimental details and the application of the proposed algorithm to diverse synthetic and real MRI images are delineated in section 4. Lastly, Section 5 remarks the concluding summary of the research (Anand et al., 2023; Uppal et al., 2023).

## 2 Materials and methods

### 2.1 Fuzzy C-means

The FCM is a widely used clustering approach that aims to divide the data into groups such that each data point has a particular degree of membership $\mu_{ik}$ , that binds the datapoint with a particular cluster with certain percentage. The membership $\mu_{ik}\text{,}$ that each data point has with the cluster center is calculated by measuring the ratio of the distance between them and others.

The process of FCM is defined by the given equation of the objective function as in Eq. (1).


(1) $$J_{FCM}=\overset{C}{\underset{i=1}{\sum }}\overset{N}{\underset{k=1}{\sum }}\mu_{ik}^{m}d{\left(x_{k},v_{i}\right)}^{2}$$

Here $\mu_{ik}$ represents membership function, $d\left(x_{k},v_{i}\right)$ represents the distance metrics between every point of the data $x_{k}$ , center of cluster $v_{i}$ and the variable m∈[1,∞] determines the amount of fuzziness. In order to satisfy the imposed probability constraint and minimize the objective function of FCM, the degree of membership and center are determined. Eq. (2) defines the imposed constraint.


(2) $$\overset{c}{\underset{i=1}{\sum }}\mu_{ik}=1,\mu_{ik}\in \left[0,1\right],0\leq \overset{N}{\underset{k=1}{\sum }}\mu_{ik}\leq N$$

where number of clusters are defined manually by the variable c.

Here Eqs (3) and (4) defines the membership matrix and cluster center as:


(3) $$\mu_{ik}=\frac{1}{\sum_{i=1}^{c}{\left(\frac{d\left(x_{k},v_{i}\right)}{d\left(x_{k},v_{j}\right)}\right)}^{\frac{2}{m-1}}}$$

(4) $$v_{i}=\frac{\sum_{k=1}^{N}\mu_{ik}^{m}x_{k}}{\sum_{k=1}^{N}\mu_{ik}^{m}}$$

The primary problem with the FCM is that it is susceptible to noise and other visual artifacts, and its objective function lacks any spatial information.

Generally, the flow of the unsupervised clustering algorithm can be given in following steps.

### 2.2 Conditional spatial fuzzy C-means

In order to remove the drawback of FCM, improved version of spatial algorithm known as csFCM was given by Pedrycz (1996). csFCM includes the conditioning aspect of the clustering mechanism as the spatial properties of an image. This conditioning aspect allows smoothening of the pixel within its specified vicinity. In csFCM, firstly $\mu_{ik}$ and $v_{i}$ are calculated as given in Eqs (3) and (4). Furthermore, spatial membership function $\mu_{ik}$ is calculated using conditional variable $h_{ik}$ as in Eq. (5).


(5) $$u_{ik}=\frac{h_{ik}}{d{\left(x_{k},v_{i}\right)}^{\frac{2}{m-1}}/\sum_{i=1}^{C}d{\left(x_{k},v_{i}\right)}^{\frac{2}{m-1}}}$$

Here $h_{ik}$ , represents an auxiliary conditional variable that determines the extent to which a pixel is associated with the specific cluster by taking into account its spatial neighborhood and is calculated as Eq. (6).

In contrast to the FCM algorithm, the csFCM algorithm introduces a conditional element into the clustering process. The algorithm factors in conditioning variables, denoted as h1, h2, …, $h_{n}$ for all pixels x1, x2, …, $x_{k}$ , respectively.


(6) $$h_{ik}=\frac{\sum_{j\in N\left(x_{k}\right)}\mu_{ik}}{R}$$

$N\left(x_{k}\right)$ is a fixed size square window having the pixel $x_{k}$ as its center and R denotes the cardinality of pixels in the neighborhood. Further the weighted membership $z_{ik}$ of csFCM and new cluster center $t_{i}$ is calculated as in Eqs (7, 8).


(7) $$z_{ik}=\frac{{\left(\mu_{ik}\right)}^{p}{\left(u_{ik}\right)}^{q}}{\sum_{i=1}^{C}{\left(\mu_{ik}\right)}^{p}{\left(u_{ik}\right)}^{q}}$$

(8) $$t_{i}=\frac{\sum_{k=1}^{N}z_{ik}^{m}x_{k}}{\sum_{k=1}^{N}z_{ik}^{m}}$$

where in Eq. (7), local $\mu_{ik}$ and global $u_{ik}$ membership values are weighted by constants p and 𝑞. These constants are used to regulate the respective importance of both the memberships for construction of the final weighted membership function and cluster center.

### 2.3 Intuitionistic fuzzy C-means

The theory of intuitionistic fuzzy sets (IFS) was introduced by Atanassov (1986). Unlike regular fuzzy sets (FS), IFS take into account a data point’s membership and non-membership values while also taking into account the presence of a third parameter, i.e., hesitation degree. In IFS, the limitation imposed on the non-membership degree is that it is not the complement of the membership value (Kang et al., 2018).

The IFS ( $S_{f})$ for dataset X can be represented as:


(9) $$S_{f}=\left\{x_{ik},\;\mu_{S}\left(x_{ik}\right),\;\lambda_{S}\left(x_{ik}\right),\;\pi_{S}\left(x_{ik}\right)\;,\;x_{ik}\;\epsilon \;X\right\}$$

In the Eq. (9), the third parameter known as hesitation degree is introduced which differentiates the FS from IFS. Here, $\mu_{S}\left(x_{ik}\right)\to \left[0,1\right],\lambda_{S}\left(x_{ik}\right)\to \left[0,1\right]$ and $\pi_{S}\left(x_{ik}\right)\to \left[0,1\right]$ represents the membership matrix, non-membership matrix and hesitation degree matrix of the data point $x_{ik}$ in an $S_{f}$ with the following condition as in Eq. (10):


(10) $$0\leq \mu_{S}\left(x_{ik}\right)+\lambda_{S}\left(x_{ik}\right)\leq 1$$

Chaira (2011) proposed novel Intuitionistic Fuzzy C-means (IFCM) approach for segmenting medical images. The IFCM introduced third parameter with the presence of membership and non-membership degree known as hesitation degree. It is calculated with the help of fuzzy membership complements using yagers complement and the sugeno complement. Dubey et al. (2016) in his research has introduced a new measure of fuzzy complement in the presence of uncertainty. The non-membership calculated using these set of fuzzy complement does not give non-membership as complement of membership. Thus, there is another factor known as hesitation degree which is $\pi_{S}\left(x_{ik}\right)=1-\mu_{S}\left(x_{ik}\right)-\lambda_{S}\left(x_{ik}\right)$ and $\pi_{S}\left(x_{ik}\right)$ is a measure of hesitation degree.

## 3 Proposed conditional spatial intuitionistic fuzzy C-means

The three drawbacks of FCM algorithm are:

- The FCM’s objective function does not integrate spatial information; it treats each pixel as an individual intensity value. Image noise, which emerges during image acquisition, can lead to altered pixel intensity values, introducing both noise and intensity inhomogeneity (Zijdenbos and Dawant, 1994). Consequently, due to FCM’s susceptibility to noise, noisy pixels tend to be misclassified in images.
- The relative distance between value of the pixel of the image and the cluster center determines the membership degree of the FCM. Pixels in close proximity to the centroid are attributed higher membership degrees, while those in distant clusters receive lower membership degrees (Hua et al., 2021). Consequently, the values of the membership becomes delicate in the presence of noise.
- Furthermore, FCM neglects to account for the presence of uncertainty or hesitation that might be inherent in real-world datasets (Ullah et al., 2023).

To some extent, csFCM proved to provide better clustering results in image segmentation. However, this algorithm does not consider the degree of hesitation or uncertainty that may exist in the real-world datasets, so the noisy pixels are not properly classified in its neighborhood. To overcome the problem of csFCM and other research work, we have proposed conditional spatial intuitionistic fuzzy C-means (csIFCM) for generating better segmentation results (Dhiman et al., 2022). In conventional FCM, the values of the non-membership degree typically complements the values of the membership degree. However, within the context of an intuitionistic approach, the non-membership degree is adjusted using fuzzy generators. This adaptation aims to effectively manage the inherent uncertainty (hesitation degree) in the dataset.

In the proposed algorithm, first we have normalized every pixel value of the image using the process of normalization in order to range the pixel value between 0 and 1. Further initial values of the number of the clusters (segments), random membership matrix, and random values of cluster centers are initialized. Then the value of the conditional spatial membership $u_{ik}^{*}$ is calculated as mentioned in Eq. (11) using spatial membership function $h_{ik}$ given by Eq. (12).


(11) $$u_{ik}^{*}=\frac{h_{ik}}{d{\left(x_{k},v_{i}\right)}^{\frac{2}{m-1}}/\sum_{i=1}^{C}d{\left(x_{k},v_{i}\right)}^{\frac{2}{m-1}}}$$

(12) $$h_{ik}=\frac{\sum_{j\in N\left(x_{k}\right)}\mu_{ik}}{R}$$

$N\left(x_{k}\right)$ is a fixed size square window having the pixel $x_{k}$ as its center and R denotes the cardinality of pixels in the neighborhood. Then, a non-membership matrix is calculated using yager’s fuzzy generator (Chaira, 2011) as given in Eq. (13).


(13) λS=1−uik∗α1α

Here α is a hyperparameter that controls the degree of hesitation. Further, we calculate the hesitation degree as Eq. (14).


(14) $$\pi_{S}\left(x_{ik}\right)=1-u_{ik}^{*}\left(x_{ik}\right)-\lambda_{S}\left(x_{ik}\right)$$

Subsequently, an conditional spatial intuitionistic membership matrix is computed by adding together the conditional membership and hesitation degree, as detailed in the Eq. (15).


(15) $${\mu^{*}}_{S}=\pi_{S}\;\left(x_{ik}\right)+u\left(x_{ik}\right)$$

Further the weighted intuitionistic membership matrix $w_{ik}$ of csIFCM and joint cluster center $g_{ik}$ is calculated as


(16) $$w_{ik}=\;\frac{{\left(\mu_{ik}^{*}\right)}^{p}{\left(u_{ik}^{*}\right)}^{q}}{\sum_{i=1}^{C}{\left(\mu_{ik}^{*}\right)}^{p}{\left(u_{ik}^{*}\right)}^{q}\;}$$

(17) $$g_{ik}=\frac{\sum_{k=1}^{N}w_{ik}^{m}x_{k}\;}{\sum_{k=1}^{N}w_{ik}^{m}\;}$$

The Eq. (16), represents the mathematical formulation of weighted membership function, where the parameters p and q are utilized to regulate the extent of intuitionistic fuzziness and the level of spatial membership. These parameters play a pivotal role in constructing the ultimate intuitionistic weighted membership function and updating the cluster centers. This $w_{ik}$ matrix finally defines the weighted membership of the pixel with the segment of an image. In regions characterized by homogeneity, the spatial function reinforces the original membership function, leaving the clustering outcome unaltered.

However, in the case of noisy pixels, which typically do not belong to the cluster, classification becomes easier through considerations of the neighboring pixels. Consequently, inaccurately classified pixels stemming from spurious clusters or noisy regions can be effectively rectified. This attribute renders the proposed csIFCM algorithm notably robust against noise and other image artifacts, ultimately enhancing the accuracy of image segmentation.

The process is repeated till the termination criterion $(w_{i+1}^{*}-w_{i}^{*}\leq \varepsilon$ ) is met.

**Table:** 


**Proposed csIFCM Algorithm** **Input:** Image X with $\left(x_{1},x_{2},x_{3}\ldots \ldots \ldots x_{k}\right)$ normalized pixel points, number of clusters c . **Output:** Weighted intuitionistic membership matrix $w_{ik}$ , joint cluster centers $g_{ik}$ Initialize Partition Matrix $\mu_{ik}$ and cluster centers $v_{i}$ , initial parameters Calculate non-membership matrix using yagers compliments, hesitation degree and intuitionistic membership matrix. for i=1ton for k=1toc Repeat for j=1,2,3… Update the conditional spatial membership matrix $u_{ik}^{*}$ and intuitionistic membership matrix $\mu_{ik}^{*}$ . Update new weighted membership $w_{ik}$ matrix and joint cluster centers $g_{ik}$ using Eqs (16) and (17) Until $(w_{i+1}^{*}-w_{i}^{*}\leq \varepsilon$ ) end end Return $w_{ik}$ and $g_{ik}\text{.}$


**Figure 1:**
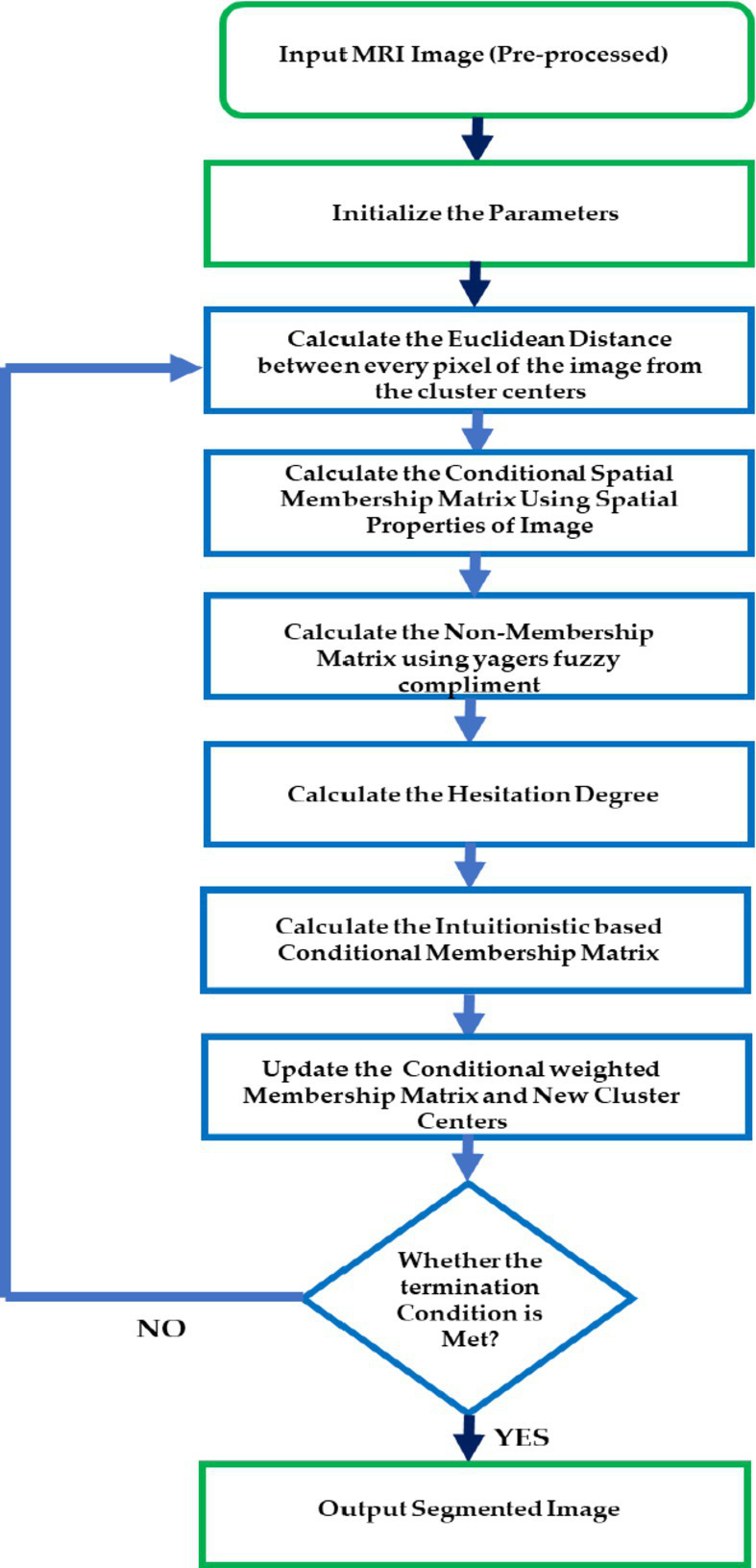
Proposed algorithm csIFCM for segmentation of MRI images.

In the proposed csIFCM algorithm, steps are executed as per the above given algorithm. Figure 1 shows the flow of the methodology.

## 4 Experiment results and discussion

Within this section, we undertake an evaluation of our proposed approach’s performance. This evaluation encompasses both synthetic images derived from the phantom dataset and actual MRI images of the human brain sourced from The Brain Atlas of Harvard Medical School, Harvard University, and the Internet Brain Segmentation Repository (Internet Brain Atlas, 2024; Internet Brain Segmentation Repository, 2024). This evaluation entails a comprehensive assessment encompassing qualitative and quantitative analyses. We evaluate the efficiency of the proposed csIFCM in comparison to the other algorithms, including FCM, IFCM, and csFCM. Notably, all clustering algorithms were implemented utilizing MATLAB (R2015a). Additionally, trial and error are used to determine the ideal values for the weighted membership function’s exponents (m, p, and q). Non-membership matrix and hesitation degree is calculated using the self-chosen alpha parameter which are used to calculate the intuitionistic membership matrix used in proposed csIFCM method.

### 4.1 Initialization of parameters

**Figure 2:**
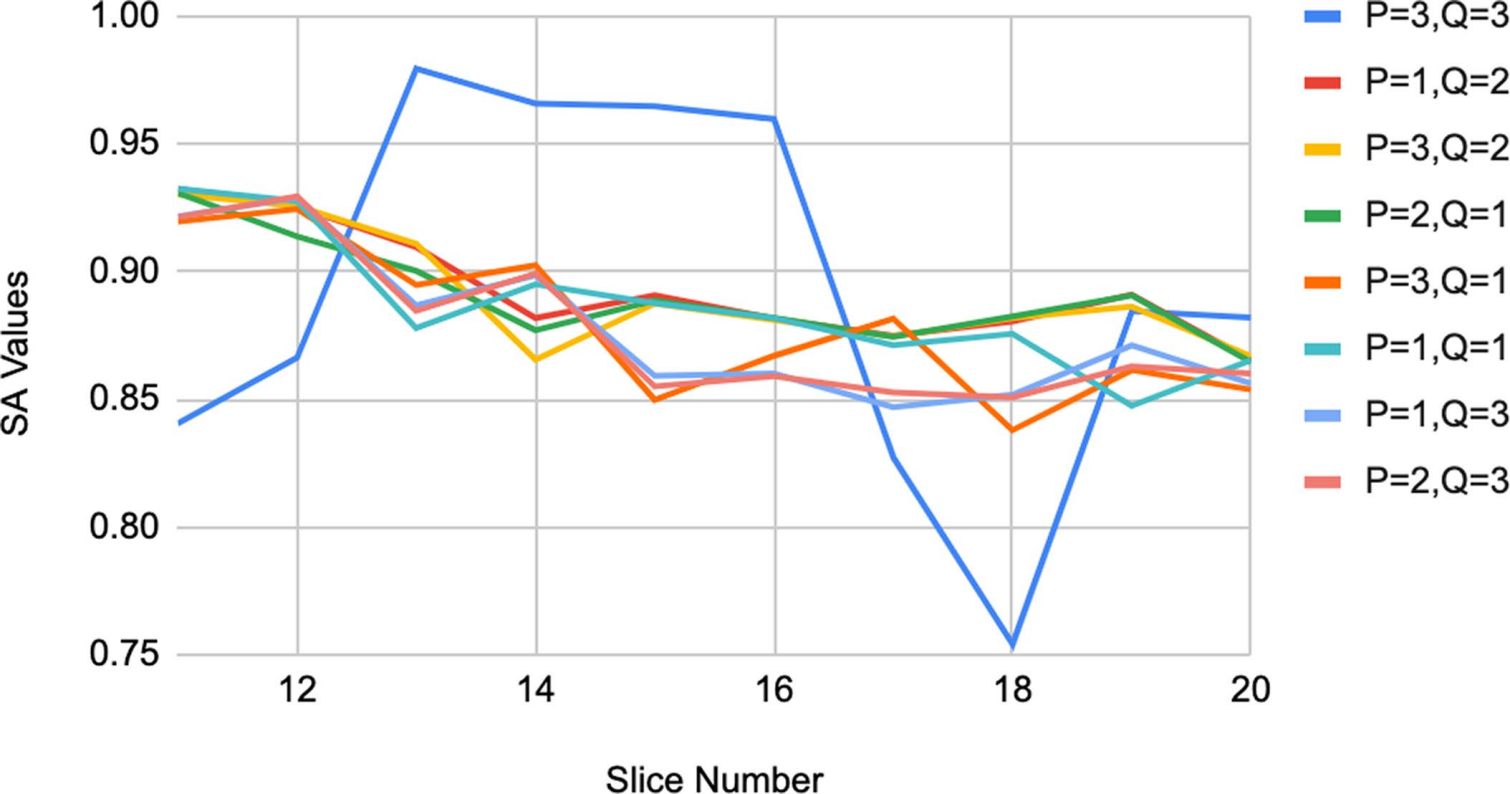
Results of segmentation accuracy on MRI images for different values of p and q.

The parameters p and q have a significant influence on the weighted membership function w and joint cluster center, thereby effecting the accuracy of csIFCM. To fix the values of p and q for csIFCM, we have calculated the segmentation accuracy of the MRI images by segmenting the image at different values of p and q (Anand et al., 2023; Uppal et al., 2023). Figure 2 represents the segmentation accuracy attained on different slices (10–20) (Internet Brain Atlas, 2024) of MRI image with respect to different values of p and q. Table 1. represents the average value of segmentation accuracy on different slices of MRI images with respect to values of p and q. It is observed from Table 1 and Figure 2 that csIFCM is giving optimal results on *p* = 1 and q = 2. The outcomes underscore the necessity for balanced emphasis on both local and global membership values during the convolution process.

**Table 1:** Segmentation accuracy of MRI images by different values of p and q for csIFCM.

p/q	1	2	3
1	0.891	**0.8975**	0.882
2	0.894	0.8972	0.882
3	0.884	0.895	0.848

Bold values represent the results of the proposed algorithm.

This equilibrium is vital for generating the ultimate intuitionistic weighted membership values and joint cluster centers, wherein equal significance is attributed to both the conditional spatial membership matrix $u_{ik}^{*}$ and the intuitionistic membership matrix $\mu_{ik}^{*}$ . Therefore, for all conducted experiments, the optimal parameter values are set as p = 1 and q = 2.

### 4.2 Qualitative and quantitative analysis

The qualitative results provide visual details about the different segments in the image. This evaluation demonstrates the algorithm’s resilience in the face of noise. To quantitatively validate the quality of segmentation, we employed various metrics, including the false negative ratio $\left(fn_{r}\right)$ , false positive ratio $\left(fp_{r}\right)$ , similarity index ( ρ) , and overall segmentation ratio. The segment’s $fp_{r}$ indicates the error arising from surplus pixels, while the $fn_{r}$ represents the inaccuracy occurs due to omitted pixels. The ρ refers to the pixels that align between the ground truth and the experimental outcomes. The segmentation accuracy (Zijdenbos and Dawant, 1994; Hua et al., 2021; Dhiman et al., 2022; Ullah et al., 2023) is characterized as the proportion of accurately classified pixels in relation to the entire pixel count within the ground truth image.


(18) $$fp_{r}=\frac{\left|S_{2}\right|-\left|S_{1}\cap S_{2}\right|}{\left|S_{1}\right|}$$

(19) $$fn_{r}=\frac{\left|S_{1}\right|-\left|S_{1}\cap S_{2}\right|}{\left|S_{1}\right|}$$

(20) $$\rho =\frac{2\left|S_{1}\cap S_{2}\right|}{\left|S_{1}\right|+\left|S_{2}\right|}$$

where $S_{1}$ and $S_{2}$ in Eqs (18)–(20) denote the pixels belonging to the ground truth value of the segment and experimental result obtained from the respective algorithm. Weighted membership function Parameters p and q have a significant influence on the weighted membership function w and joint cluster center g , thereby affecting the segmentation accuracy.

#### 4.2.1 Synthetic image of phantom

A synthetic image was produced utilizing the built-in MATLAB function, phantom(). This function generates an image of a head phantom designed for assessing the numerical precision of various algorithms. The resulting grayscale intensity image comprises a prominent large ellipse symbolizing the brain, within which multiple smaller ellipses are embedded to symbolize distinct features within the brain. The quantitative outcomes of the segmentation attained by the various methods are displayed in Table 2. Table 2 indicates that the clustering method csIFCM, which is being proposed, is doing better in terms of overall segmentation accuracy.

**Table 2:** Quantitative measures of phantom image.

Algorithm	Segment	Similarity index	False positive ratio	True positive ratio	Overall segmentation accuracy
FCM	1	0.8305	0.0008	0.7106	0.906
	2	0.9687	0.0008	0.9324	
	3	0.9596	0.0097	0.9326	
	4	0.9598	0.0097	0.9329	
IFCM	1	0.4574	2.3729	1	0.8972
	2	0.9862	0.0131	1	
	3	0.9862	0.0131	1	
	4	0.9561	0.0101	0.9268	
csFCM	1	1	0	1	0.9187
	2	0.8601	0.0007	0.7544	
	3	0.9723	0.0018	0.9420	
	4	0.9651	0.0093	0.9423	
csIFCM	1	0.9398	0.0015	0.8877	**0.9631**
	2	0.9865	0.0013	0.9736	
	3	0.9866	0.0013	0.9737	
	4	0.9843	0.0047	0.9738	

Bold values represent the results of the proposed algorithm.

The overall segmentation ratio should have a value of 1, with a greater value being better, for the better outcome. The suggested algorithm has a higher overall segmentation ratio (0.9631) than the other algorithms.

**Figure 3:**
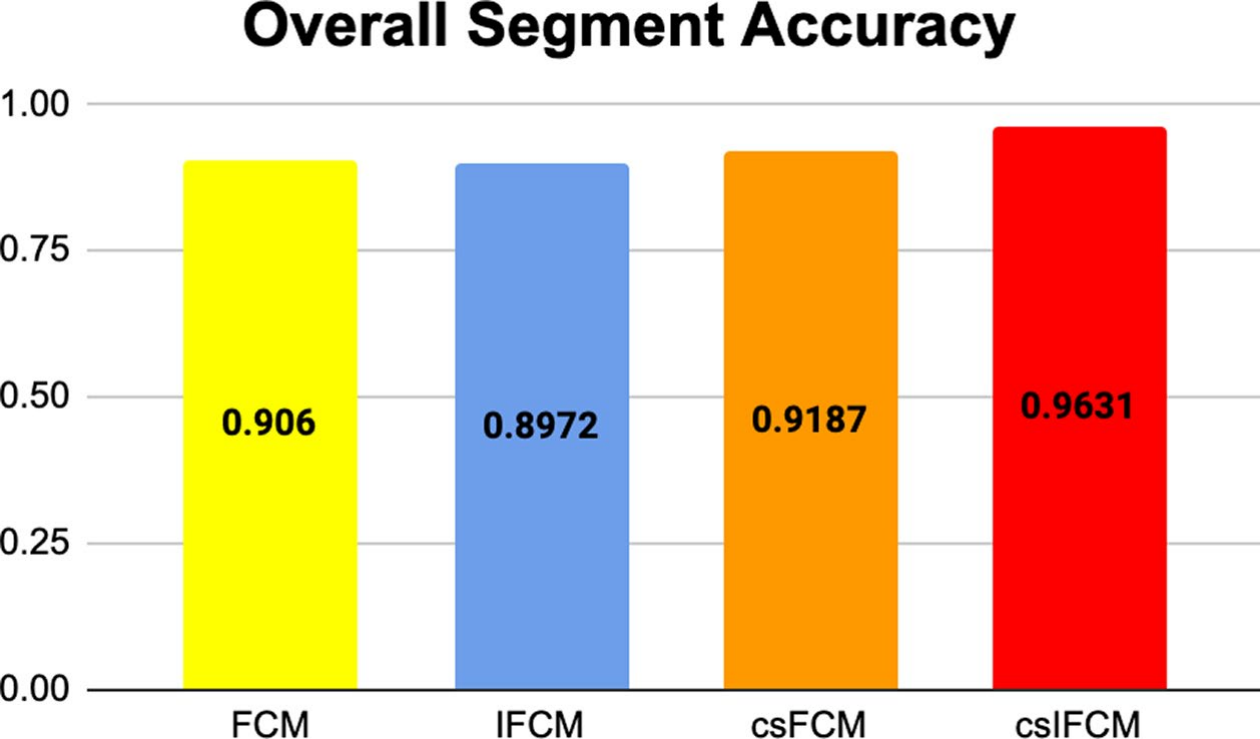
Results of overall segmentation accuracy of different algorithms for phantom image.

**Figure 4:**
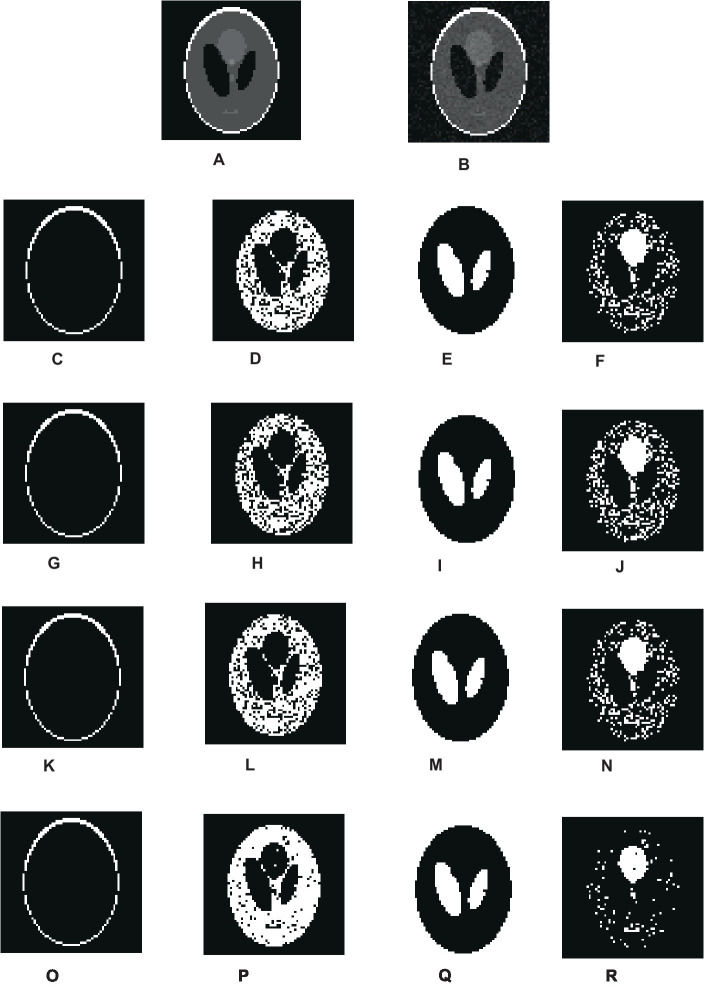
Visual results of segmentation on synthetic image of phantom. **(A)** Phantom image. **(B)** Noisy images. **(C–F)** Results of FCM. **(G–J)** Results of IFCM. **(K–N)** Results of csFCM. **(O–R)** Results of csIFCM.

Figure 3 shows the overall segmentation accuracy achieved after segmentation and the proposed algorithm proved to give maximum accuracy. Figure 4 shows qualitative results of the phantom image which has been segmented into four segments representing different regions of interest in the image.

To show the effectiveness of the algorithm against noise, we have embedded an image with 0.01% of Gaussian noise. Figures 4A,B depict the original and noisy images, respectively. The noisy image’s segmented sections, produced by the FCM algorithm, are displayed in Figures 4C–F. Figures 4G–J displays the IFCM result. The results of csFCM are displayed in Figures 4K–N, and the suggested csIFCM algorithm produces results with more qualitative accuracy and greater noise resilience in Figures 4O–R.

The second experimentation involved the analysis of two real MRI brain images sourced from references (Internet Brain Atlas, 2024; Internet Brain Segmentation Repository, 2024). Specifically, the experiments were conducted on T-1 weighted axial slices ranging from slide numbers 85–115 (Internet Brain Atlas, 2024), considering noise. The primary objective was the segmentation of images into four distinct segments. It’s noteworthy that these images are not provided with the ground truth value.

#### 4.2.2 Real MR brain image

To quantify the results, a comparative analysis was performed by introducing 2% Gaussian noise to the original images and subsequently comparing the outcomes. This approach was employed as the ground truth values of the given data set was not provided. Specifically, the algorithm’s performance was scrutinized by examining the variation in results when noise was added to the original images, thereby checking its effectiveness in handling noisy scenarios.

**Figure 5:**
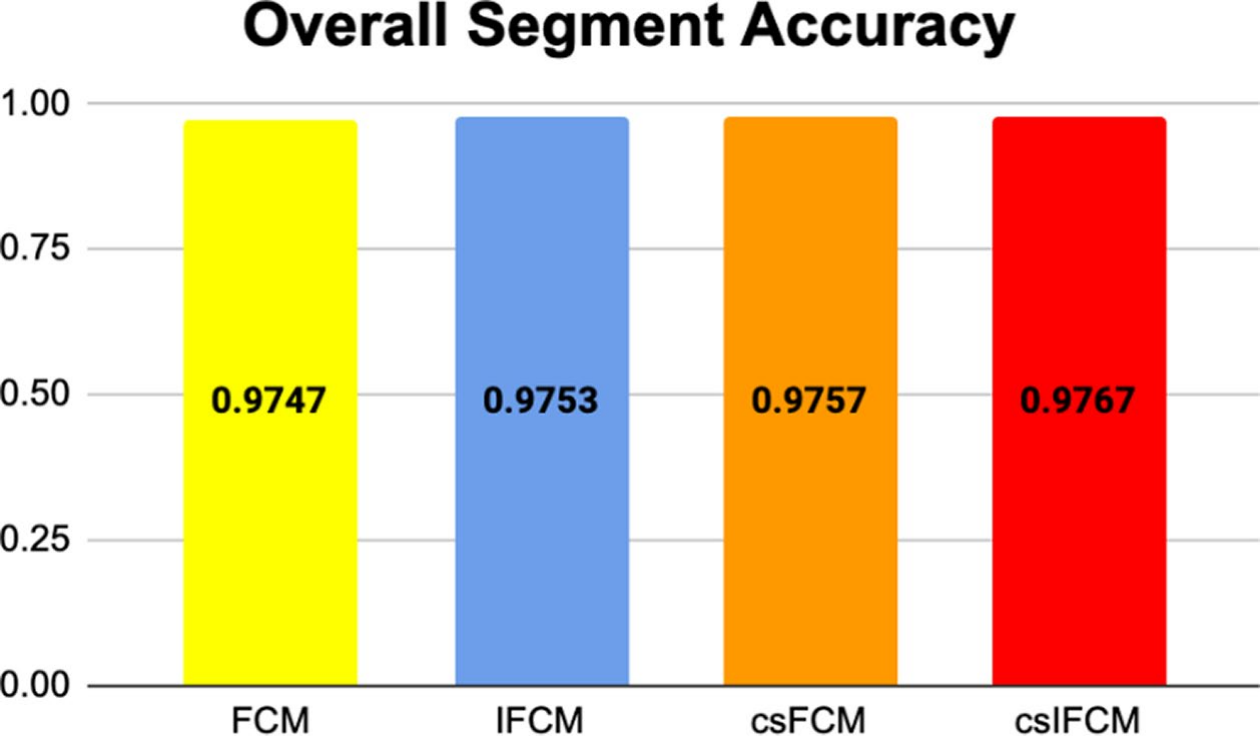
Plot showing overall segmentation accuracy for different algorithms.

Table 3 shows the quantitative results produced by the implementation on T-1 weighted MRI axial image by FCM, IFCM, csFCM, and proposed csIFCM. Figure 5 shows the overall segmentation accuracy over all the segments and thus indicates that the proposed csIFCM outperforms all the other algorithms and validate the effectiveness of the proposed csIFCM over the other techniques under comparison.

**Table 3:** Quantitative measures of T-1 weighted MRI axial image in the presence of noise.

Algorithm	Segment	Similarity index	False positive ratio	True positive ratio	Overall segmentation accuracy
FCM	1	0.9263	0.0045	0.9097	0.9747
	2	0.9825	0.0062	0.9919	
	3	0.9910	0.0078	0.9902	
	4	0.9902	0.0092	0.9900	
IFCM	1	0.9265	0.1389	0.9883	0.9753
	2	0.9837	0.0808	0.9488	
	3	0.9915	0.0643	0.9191	
	4	0.9907	0.0109	0.9897	
csFCM	1	0.9268	0.0829	0.9106	0.9757
	2	0.9845	0.1662	0.9320	
	3	0.9915	0.0682	0.9935	
	4	0.9907	0.1260	0.9819	
csIFCM	1	0.9300	0.0592	0.9206	**0.9767**
	2	0.9880	0.0627	0.9330	
	3	0.9917	0.0093	0.9927	
	4	0.9909	0.0108	0.9908	

Bold values represent the results of the proposed algorithm.

**Figure 6:**
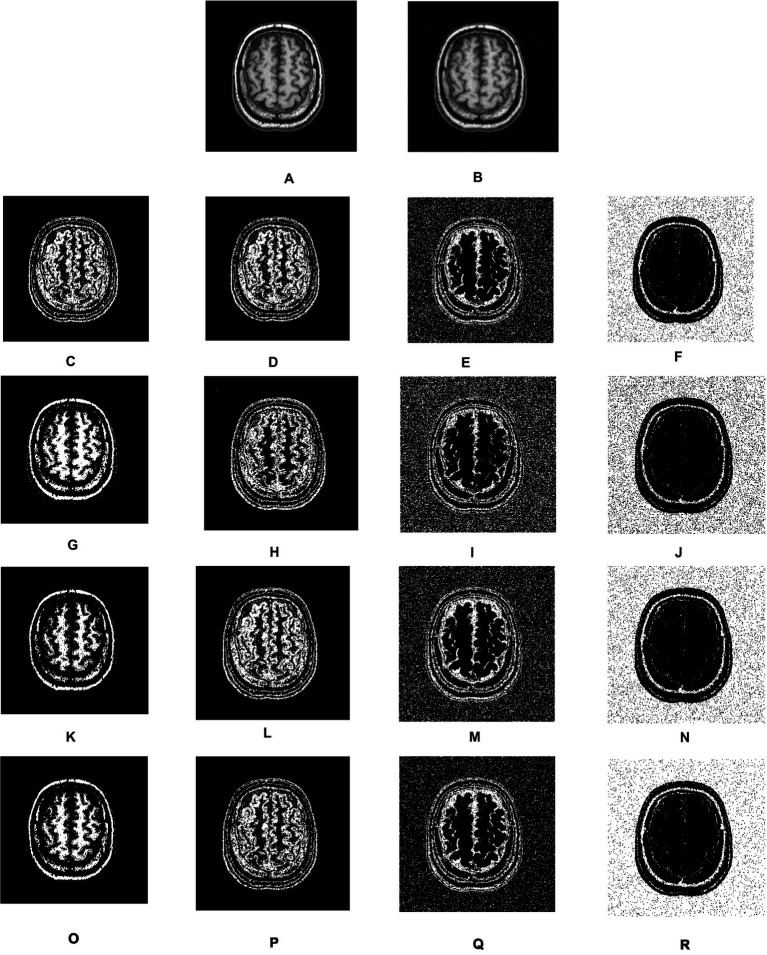
Visual results of real T-1 MR axial slice (10) scan. **(A)** Real image. **(B)** Noisy images. **(C–F)** Segmentation results of FCM. **(G–J)** Segmentation results of IFCM. **(K–N)** Segmentation results of csFCM. **(O–R)** Segmentation results of csIFCM.

Figure 6 shows the qualitative result of segmentation generated by FCM, IFCM, csFCM, and proposed csIFCM. A deep investigation reveals a better visualization of details in the MRI image in Figures 6O–R, where the proposed csIFCM is showing more robust results and demonstrates its superiority as compared to other algorithms. The csIFCM algorithm exhibits robustness in the presence of noise, retaining well-defined image edges and preserving a greater amount of image details.

**Figure 7:**
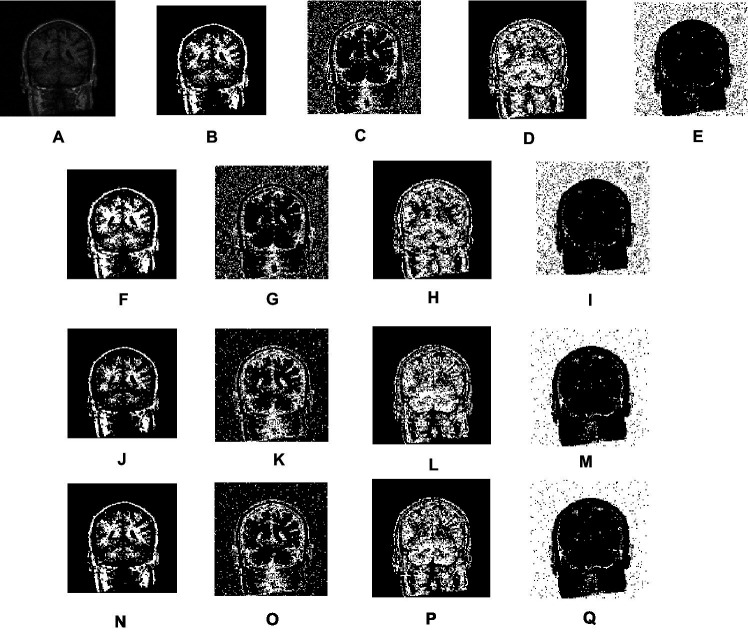
Visual results of real MRI scan. **(A)** Real images. **(B–E)** Segmentation results of FCM. **(F–I)** Segmentation results of IFCM. **(J–M)** Segmentation results of csFCM. **(N–Q)** Segmentation results of csIFCM.

Second, we employed actual MRI brain scans from the Internet Brain Segmentation repository (Internet Brain Atlas, 2024), which also provides manually segmented (ground truth values) data for verifying the outcomes of novel segmentation techniques. We have segmented real T1-weighted MRI brain images in 2D axial slices 10 to 20 using FCM, IFCM, csFCM, and the proposed csIFCM algorithms. Figure 7 displays the segments obtained for slice 10. Figure 7A shows the original image, which will be segmented into 4 clusters. Figures 7B–E shows the segmentation results obtained by FCM, Figures 7F–I showcases the segmentation results of IFCM, Figures 7J–M shows the segmentation results of csFCM and Figures 7N–Q shows the segmentation results of proposed csIFCM. The outcomes clearly confirm the superior performance of the proposed algorithm compared to the other algorithms under comparison. The csIFCM algorithm exhibits robustness in the presence of noise, retaining well-defined image edges and preserving a greater amount of image details.

In quantitative terms, we have computed both the true positive rate (TPR) and the false positive rate (FPR) (Adhikari et al., 2015; Arora and Tushir, 2019). This calculation facilitates the portrayal of a satisfactory balance between these two performance metrics


(21) $$TPR=\frac{TP}{P}$$

(22) $$FPR=\frac{FP}{P}$$

In this particular context, P symbolizes the count of positive instances, while N signifies the count of negative instances. When a prediction yields a positive result and the actual value is likewise positive, it falls under the category of true positive. Conversely, when a prediction yields a positive result but the actual value is negative, it is classified as a false positive. Table 4 shows the average value of the results obtained by the different segments for all the algorithms. The results indicate that the proposed csIFCM algorithm gives better results as compared to the FCM, IFCM, csFCM. The csIFCM gives maximum segmentation accuracy.

**Table 4:** Quantitative measures of T-1 weighted MR axial image of slices 10–20.

Algorithm	Similarity index	True positive ratio	False positive ratio	Segmentation accuracy
FCM	0.8866	0.8655	0.120	0.8713
IFCM	0.8883	0.8778	0.114	0.8753
csFCM	0.8977	0.8919	0.093	0.8808
csIFCM	**0.9100**	**0.9002**	**0.083**	**0.8975**

Bold values represent the results of the proposed algorithm.

**Figure 8:**
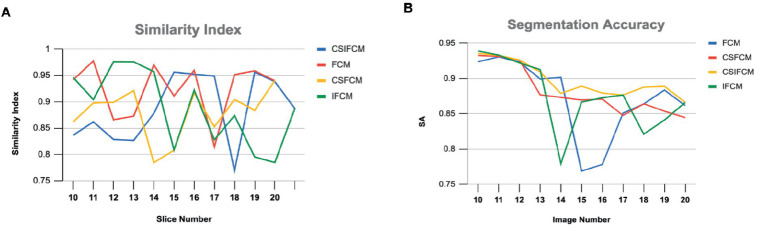
**(A)** Similarity Index of slices (10–20) of T-1 weighted MR axial image of FCM, IFCM, csFCM, and csIFCM. **(B)** Segmentation Accuracy of slices (10–20) of T-1 weighted MR axial image of FCM, IFCM, csFCM, and csIFCM.

Figure 8 shows the plot of similarity index achieved from FCM, IFCM, csFCM and proposed csIFCM algorithm on image slices (10–20) of the T-1 weighted MR brain images. The overall segmentation accuracy and similarity index is represented for each slice. These results indicate that the proposed csIFCM technique is performing better in Figure 8A, with the highest average value of similarity index for each slice and in Figure 8B, the value of segmentation accuracy is better in each slice as compared to other algorithms and demonstrate its superiority over the FCM, IFCM, csFCM.

##### 4.2.2.1 Time complexity analysis

To compute the similarity between each pixel requires $O\left(n*c*d\right)$ operations, where n is the number of data points, c is the number of clusters and d is the dimensionality of the data. To update the membership degrees for each data point, this typically requires $O\left(n*c\right)$ operations. In intutiotionistic approach, non-membership is calculated using membership matrix in constant time. The overall time complexity of the standard IFCM is $O\left(n*c*d*I\right)$ where I is the number of iterations required for convergence. Incorporating the conditional spatial information, includes calculation of spatial information in the time complexity of $O\left(n^{2}\right)\text{.}$ Further, to calculate the weighted spatial membership matrix involves constant time. Therefore, the overall time complexity of incorporating conditional spatial information in csIFCM can be approximated as $O\left(n*c*d*I+N*K\right)$ where N is the number of datapoints in the spatial neighborhood of each point and K is the computational cost of processing the conditional spatial information.

#### 4.2.3 Limitation of the conditional spatial intuitionistic fuzzy C-means

The limitation of csIFCM is its senstivity to choice of initial parameters. The initial parameters are selected randomly which may result in the increase of convergence time. The performance of csIFCM depends on how the spatial neighborhood is defined and selection of the size of neighborhood window along with the pixel under consideration which defines spatial relationship criterion. Defining a suitable spatial neighborhood enables the accurate capture of spatial structure, leading to optimal segmentation results.

Additionally, csIFCM experiences increased computational time complexity compared to IFCM due to the incorporation of spatial information and the calculation of the weighted membership matrix. The computation of spatial relationships or distances between data points adds an additional overhead to the clustering process, which can be significant, especially for large datasets or complex spatial structures.

## 5 Conclusion and future scope

This work introduces a novel algorithm named Conditional Spatial Intuitionistic Fuzzy C-means (csIFCM) for the segmentation of MRI images. By incoporating both local gray-level and spatial information through the introduction of a conditional spatial variable, csIFCM addresses the limitations of existing methods, particularly in scenarios involving noise and intensity inhomogeneity. Our experiments encompass synthetic phantom images, as well as real and simulated MRI brain images. We can explore kernel metrics that will help to segment non-linear data with higher accuracy.

## Data availability statement

Publicly available datasets were analyzed in this study. This data can be found at: http://www.med.harvard.edu/aanlib/cases/caseNA/pb9.htm; http://www.cma.mgh.harvard.edu/ibsr/.

## Ethics statement

Written informed consent was not required from the individual(s) for the publication of any potentially identifiable images or data included in this article as the dataset is taken from a public repository.

## Author contributions

JA: Conceptualization, Writing – original draft. GA: Writing – review & editing, Software, Project administration, Funding acquisition, Supervision, Methodology. AN: Writing – review & editing, Methodology, Investigation, Data curation, Conceptualization. MT: Writing – review & editing, Project administration, Formal analysis. TS: Writing – review & editing, Validation, Resources, Project administration. DG: Writing – review & editing, Supervision, Methodology, Investigation. SK: Writing – review & editing, Validation, Project administration, Formal analysis.
